# Investigation of Sex Differences in the Microglial Response to Binge Ethanol and Exercise

**DOI:** 10.3390/brainsci7100139

**Published:** 2017-10-24

**Authors:** Emily A. Barton, Cassandra Baker, J. Leigh Leasure

**Affiliations:** 1 Department of Psychology, University of Houston, Houston, TX 77204, USA; eabarton3@uh.edu (E.A.B.); cabaker5@uh.edu (C.B.); 2 Department of Biology & Biochemistry, University of Houston, Houston, TX 77204, USA

**Keywords:** alcohol, hippocampus, medial prefrontal cortex, microglial priming, MHC II

## Abstract

The female brain appears selectively vulnerable to the neurotoxic effects of alcohol, but the reasons for this are unclear. One possibility is an exaggerated neuroimmune response in the female brain, such that alcohol increases microglia number and reactivity to subsequent stimuli, such as exercise. It is important to better characterize the interactive neural effects of alcohol and exercise, as exercise is increasingly being used in the treatment of alcohol use disorders. The present study compared the number of microglia and evidence of their activation in alcohol-vulnerable regions of the brain (medial prefrontal cortex and hippocampus) in male and female rats following binge alcohol and/or exercise. Binge alcohol increased microglia number and morphological characteristics consistent with their activation in the female brain but not the male, regardless of exercise. Binge alcohol followed by exercise did increase the number of MHC II+ (immunocompetent) microglia in females, although the vast majority of microglia did not express MHC II. These results indicate that binge alcohol exerts sex-specific effects on microglia that may result in enhanced reactivity to a subsequent challenge and in part underlie the apparent selective vulnerability of the female brain to alcohol.

## 1. Introduction

Research in the field of alcohol has historically focused on men, with women receiving comparably little representation, due to men having a higher risk for developing problematic drinking [1,2]. However, the prevalence of alcohol use disorders (AUD) in young women is increasing [3,4]. Moreover, there is evidence suggesting that women are more vulnerable to the physical consequences of excessive alcohol consumption [5,6,7,8], including the neurotoxic effects [6,9]. Some human studies have shown larger reductions in hippocampal volume, increased volumetric loss of grey and white matter, and reduced corpus callosum size in the brains of alcoholic women compared to men [6,8,9,10,11,12] although other studies have shown no sex differences in alcohol-induced brain damage [13,14]. Animal models of alcohol-induced brain damage are advantageous in that they enable alcohol dose and length of administration to be standardized between males and females. Using a 4-day binge model of alcohol-induced neurodegeneration, we have shown that female rats have a significant loss of dentate gyrus granule neurons that is not seen in males, as well as an acquisition deficit in learning the location of the platform in the Morris water maze [15]. This finding, coupled with findings from clinical populations, suggests that the female brain is specifically vulnerable to the neurotoxic effects of alcohol in both rats and humans. The mechanisms underlying the apparent vulnerability of the female brain to the damaging effects of alcohol have not been established, however sex differences in innate immunity may be a contributing factor.

The immune response in females is more exaggerated than in males, and women have a higher risk of developing autoimmune diseases and chronic pain conditions compared with men [16,17,18,19,20]. Specifically, there is a higher incidence of women developing Alzheimer’s disease [21,22], multiple sclerosis [23,24], migraines [25], fibromyalgia [26,27,28], and osteoarthritis [29]. Given the involvement of neuroinflammation in these disorders, research investigating the female vulnerability to them has begun to examine sexual dimorphisms in microglial morphology, number, and activity [17,30]. Indeed, there are sex differences in microglia number and function that begin in development [31]. In adulthood, female rats have a larger subset of microglia with thicker processes, indicative of a partially activated or primed state [16,32,33,34]. Microglia priming occurs when an initial stimulus shifts the microglia to a state that is more likely to respond to a second challenge, which results in a prolonged and exaggerated response to subsequent homeostatic disturbances [35]. This means primed microglia are more susceptible to a secondary inflammatory stimulus, as priming both decreases the response threshold and increases the pro-inflammatory response. This higher proportion of microglia kept at a more activated state in females could potentially contribute to enhanced neuroimmune responses.

Given the link between microglia and aspects of alcohol-induced neurotoxicity [36,37], a higher percentage of primed microglia in females could be a potential mediator of sex differences in the neural response to alcohol. The majority of studies examining the effects of alcohol on microglia have used only male animals, yet given the known sex differences in neuroimmune disorders, the microglial response to alcohol may be markedly different in females. Furthermore, if alcohol is indeed a priming stimulus for microglia [38,39], we would expect females to have more primed microglia and to launch a larger response to secondary challenges. We postulate that exercise may represent a secondary challenge, as we have previously found that 4 weeks of exercise after binge alcohol resulted in a drastic reduction in microglia number in the medial prefrontal cortex (mPFC) of female rats [40], in contrast to alcohol naïve animals that exercised, the latter of which showed an increase in mPFC microglia. Moreover, the microglia in the alcohol-exposed animals that exercised displayed morphological characteristics consistent with partial activation or priming. However, the microglia in the alcohol naïve animals that exercised did not show any signs of activation. These data suggest that microglia in the recently alcohol-exposed brain respond differently to exercise, possibly due to binge alcohol acting as a priming stimulus. 

Whether there are sex differences in the microglial response to alcohol and/or exercise has not been investigated. Therefore, the current set of experiments examined the differential response of microglia to binge alcohol and exercise in male and female rats in the mPFC and hippocampus. These regions were chosen because they are both vulnerable to alcohol [11,41,42] and responsive to exercise [43,44]. Microglia number, morphology, and expression of MHC II were assessed. Upregulation of MHC II allows microglia to act as antigen presenting cells (APCs) in the CNS and elicit T-cell activation [45,46]. Additionally, microglia expressing MHC II produce an exaggerated inflammatory response by upregulating the pro-inflammatory cytokine IL-1β following activation [47]. Moreover, MHC II appears to be a hallmark of microglial priming and is often used as a marker of primed microglia [47,48,49,50,51,52]. We expected binge alcohol to prime microglia to be more responsive to exercise, resulting in increased microglia with primed morphology and MHC II expression. Additionally, we hypothesized that this effect would be more marked in females.

## 2. Materials and Methods

### 2.1. Animals

All experimental procedures were conducted in accordance with the Guide for the Care and Use of Laboratory Animals of the National Institutes of Health. The relevant animal protocol was approved by the University of Houston Institutional Animal Care and Use Committee (protocol number 16-013). Forty-eight Long Evans rats (Harlan Sprague, Dawley, IN, USA), 24 male (293 to 345 g) and 24 female (220 to 264 g) and aged 9 weeks at the beginning of the experiment, were randomly divided into 4 groups to examine the effects of Diet (Control or Binge Ethanol) and Activity (Sedentary or Exercise). The groups were coded as follows: control, sedentary (CON); control, exercise (CEX); binge, sedentary (BIN); and binge, exercise (BEX, see Table 1). Following arrival, same-sex rats were housed 2–3 per cage and allowed to acclimate for 7 days to the vivarium conditions, which included a reversed light/dark cycle (lights off at 9:00/on at 21:00) and *ad libitum* food and water. All rats were gently handled daily during this time in order to acclimate them to the experimenters and make them receptive to oral gavage.

### 2.2. Alcohol Administration Paradigm

During the 4-day binge procedure, food was removed from both control and binged animals, but water was always available. All animals were given an ethanol diet (25% ethanol *w*/*v* in vanilla Ensure^TM^; Abbot Laboratories, Columbus, OH, USA), or an isocaloric control diet (dextrose with vanilla Ensure^TM^) every eight hours for four days by intragastric gavage, using a paradigm modified from Majchrowicz [53]. The initial dose was 5 g/kg; every additional dose was determined based on body weight and a 6-point scale of behavioral intoxication (Table 2), such that the more intoxicated animals received less alcohol, and vice versa. Blood ethanol concentration (BEC) was determined from saphenous vein samples taken 90 min after the 7th dose. Samples were centrifuged and serum extracted and stored at −20 °C. BECs were determined using an AM1 Analyzer based on external standards (Analox, Waltham, MA, USA). Withdrawal symptoms were monitored every 30 min for 16 h beginning 10 h after the last dose of alcohol. Withdrawal behavior was scored using a 12-point scale modified from Majchrowicz [53], with 0 indicating the lowest severity and 4 indicating the highest severity (Table 3).

### 2.3. Voluntary Exercise

On the 7th day following the last dose of alcohol or isocaloric diet, rats in the exercise groups were given voluntary access to exercise wheels for 5 h a day for 11 days. The exercise wheels were equipped with counters that recorded running distance, speed and time. Animals in the sedentary groups remained in their home cages. During the exercise period, all animals had access to rat chow and water *ad libitum*. 

### 2.4. Tissue Processing

Animals were overdosed with anesthetic and intracardially perfused with cold saline, followed by 4% paraformaldehyde until the upper body was stiff. Brains were removed, post-fixed, and stored in 30% sucrose until they were cut into 50 µm coronal sections on a freezing sliding microtome (Leica, Bannockburn, IL, USA). Tissue sections containing the mPFC were collected in a 1:6 series and sections containing the hippocampus were collected in a 1:12 series. Free-floating sections were washed in tris-buffered saline (TBS) three times for 10 min each and then quenched in 3% H_2_O_2_ to exhaust endogenous peroxidase activity. Sections were rinsed of H_2_O_2_ and then blocked in TBS containing donkey serum and triton-X for 1 h to reduce non-specific binding. Following blocking, sections were left to incubate in the primary antibody (rabbit anti-iba1, Wako Chemicals, Cape Charles, VA, USA; 1:10,000 or mouse anti-MHC II, Bio-Rad, Hercules, CA, USA, 1:50) for 72 h at 4 °C. Next, sections were washed 2 times for 15 min each in TBS and then incubated in the secondary antibody (biotinylated donkey anti-rabbit or anti-mouse, Jackson ImmunoResearch, West Grove, PA, USA, 1:250) overnight at room temperature. Following an additional three 10-min TBS washes, the sections were incubated in ABC solution (Vector Labs, Burlingame, CA, USA) for 1 h and then given an additional three rinses before treatment with diaminobenzidine (DAB). Finally, sections were given a final four TBS rinses, mounted on gelatinized slides, and coverslipped using Permount. 

### 2.5. Quantification of Microglia

**Figure 1:**
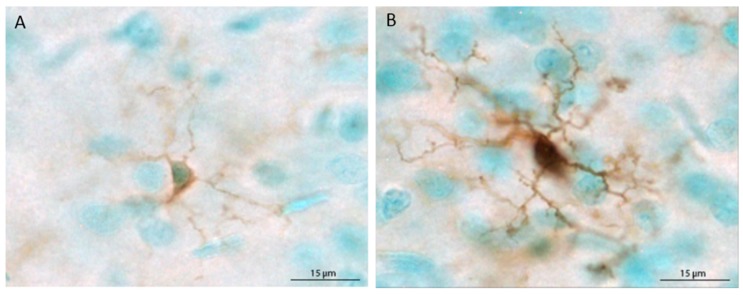
Depiction of the morphological differences between ramified (unchallenged) (**A**) and primed (**B**) microglial cells. In the current study, microglia were classified as primed when the cell body was large and completely, darkly stained and processes were darkly stained and thick.

The populations of Iba1+ cells in the mPFC and hippocampus were estimated using the optical fractionator method applied via an automated stereology system (StereoInvestigator, MicroBrightField, Williston, VT, USA). Using a Nikon Eclipse 80i upright microscope, the region of interest was traced using the 4× objective, and cells were counted within two-dimensional counting frames. The average mounted section thickness was approximately 37 µm, thus, top and bottom guard zones were set at 5 µm each, for an optical dissector height of 27 µm. For the mPFC, Iba1+ cells were counted in every sixth section in a single hemisphere, beginning at Bregma 3.20 mm and ending at Bregma 2.20 [54], using a counting frame size of 80 × 80 µm and a grid size of 400 × 400 µm. For the hippocampus, Iba1+ cells were counted in every 12th section in a single hemisphere beginning at Bregma −1.80 and ending at Bregma −6.04, using a counting frame size of 40 × 40 µm and a grid size of 200 × 200 µm. Microglia were classified as ramified (thin, lightly stained processes and small, lightly stained cell bodies) or primed (thick, darkly stained processes and large cell bodies that were completely, darkly stained) based on prior reports [34,35,48,55]. Representative ramified and primed microglia are shown in Figure 1. Each primed microglial cell within a counting frame was denoted with a second marker, which allowed for obtaining population estimates of both total microglia and primed microglia. Because very few cells were labeled with MHC II, stereological quantification was not possible. Therefore, MHC II+ cells are reported as the mean number of cells per section for each brain region. 

### 2.6. Statistical Analyses

Behavioral intoxication scores and withdrawal scores are ordinal data and were therefore analyzed using nonparametric independent samples Mann–Whitney U tests to compare group medians. Independent samples *T* tests were used for the dose and BEC data. Exercise data were analyzed using repeated measures ANOVA. Factorial 2 × 2 × 2 ANOVAs (Diet × Activity × Sex) were used to analyze microglia populations in the mPFC and hippocampus. Post hoc Tukey HSD pairwise comparisons were used when appropriate. For all statistical analyses, a *p* value of less than 0.05 was deemed significant. 

## 3. Results

### 3.1. Intoxication and Exercise Data 

The binge data for males and females are presented in Table 4. Following withdrawal, one male died unexpectedly. The median intoxication score for males was higher than females (*p* = 0.01), which resulted in a lower median dose of alcohol for males compared to females (*p* < 0.001). However, there were no significant sex differences in BEC, median withdrawal, or peak withdrawal. The exercise data for males and females are presented in Table 5. Females ran more per day than males (*p* < 0.001) and had a higher cumulative distance (*p* < 0.001). There was no sex difference in peak speed. 

### 3.2. Microglia Number and Morphology in the Medial Prefrontal Cortex 

**Figure 2:**
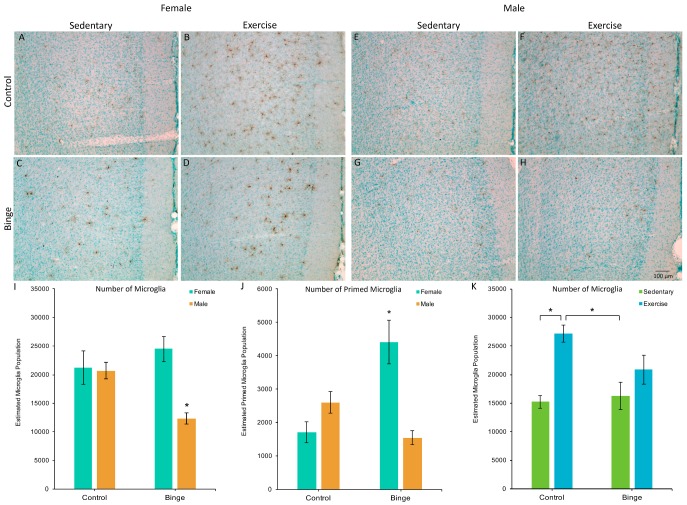
The effects of binge alcohol and exercise on microglia in the medial prefrontal cortex (mPFC) of females (**A**–**D**) and males (**E**–**H**); images of Iba1 staining in the mPFC captured at 10×. Binged males had fewer microglia in the mPFC compared to all other groups (**I**). There was a binge-induced suppression of exercise-induced microglial plasticity (**K**), as exercise increased microglia numbers in control, but not binged animals. For morphologically primed microglia (**J**), binged females had more compared to all other groups. * *p* < 0.05.

A factorial ANOVA comparing the number of microglia in the mPFC across Diet, Activity, and Sex did not reveal a significant three-way interaction (*F*(1,39) = 1.48, *p* = 0.23). However, the Diet × Sex interaction was significant (*F*(1,39) = 17.06, *p* < 0.001, Figure 2A). Post hoc pairwise comparisons showed that BIN males had fewer microglia compared to BIN females (*p* < 0.001), CON males (*p* = 0.03), and CON females (*p* = 0.01). The Diet × Activity interaction was also significant (*F*(1,39) = 6.74, *p* = 0.01, Figure 2B). Post hoc pairwise comparisons showed that CEX animals had more microglia than CON (*p* < 0.001) and BIN animals (*p* = 0.002). 

A factorial ANOVA comparing the number of microglia with a primed morphology in the mPFC across Diet, Activity, and Sex did not reveal a significant three-way interaction (*F*(1,39) = 3.43, *p* = 0.07). However, the Diet × Sex interaction was significant (*F*(1,39) = 30.71, *p* < 0.001, Figure 2C). Post hoc pairwise comparisons showed that BIN females had more morphologically primed microglia compared to BIN males (*p* < 0.001), CON females (*p* < 0.001), and CON males (*p* = 0.02). These data indicate a sex difference in the morphological response of microglia to binge alcohol, with females exhibiting increased microglial priming compared to males.

### 3.3. Microglia Number and Morphology in the Hippocampus

**Figure 3:**
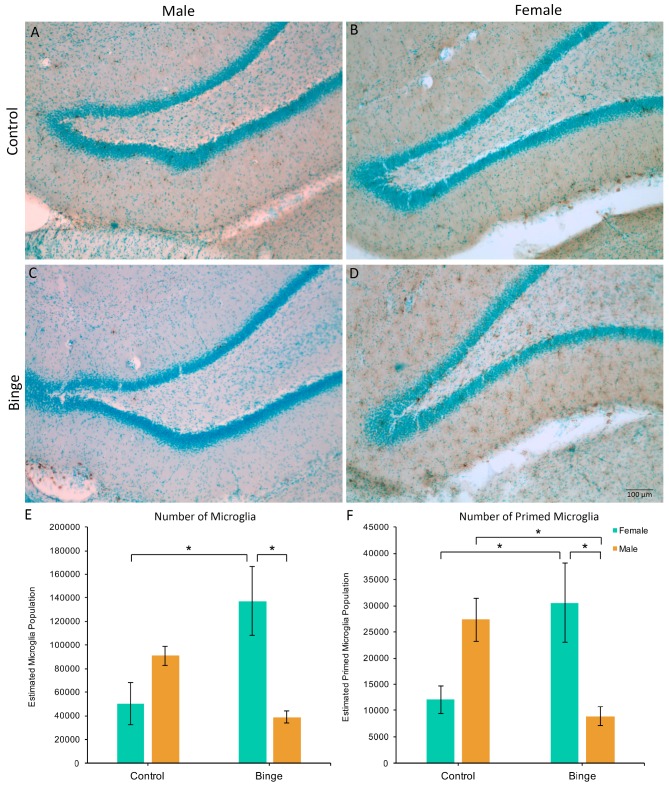
The effects of binge alcohol and exercise on microglia in the hippocampus of males (**A**,**C**) and females (**B**,**D**). Binge alcohol increased the number of microglia in females compared to female controls and binged males (**E**). Binge alcohol increased the number of morphologically primed microglia in females, but decreased it in males (**F**). * *p* < 0.05.

Similar to the number of microglia in the mPFC, a factorial ANOVA comparing the number of microglia in the hippocampus across conditions did not show a significant three-way interaction (*F*(1,39) = 0.39, *p* = 0.54), however, it did reveal a Diet × Sex interaction (*F*(1,39) = 15.45, *p* < 0.001, Figure 3A). Post hoc pairwise comparisons showed that BIN females had more microglia in the hippocampus compared to BIN males (*p* = 0.003) and CON females (*p* = 0.008). Of note, there was a main effect of Activity (*F*(1,39) = 4.44, *p* = 0.04), with exercise increasing microglia number in the hippocampus compared to sedentary animals. However, there were no interactive effects of exercise. Additionally, peak speed of exercise positively correlated with the number of hippocampal microglia in females (Pearson’s *R* = 0.61, *p* = 0.03), but not males (Pearson’s *R* = −0.29, *p* = 0.40).

The Diet × Activity × Sex interaction for the number of primed microglia in the hippocampus was not significant (*F*(1,39) = 0.81, *p* = 0.37), however, there was a significant Diet × Sex interaction (*F*(1,39) = 17.90, *p* = 0.001, Figure 3B). Post hoc pairwise comparisons showed that BIN females had more morphologically primed microglia compared to BIN males (*p* = 0.01) and CON females (*p* = 0.03). Additionally, BIN males had fewer primed microglia compared to CON males (*p* = 0.04).

### 3.4. MHC II Expression in the Medial Prefrontal Cortex and Hippocampus

**Figure 4:**
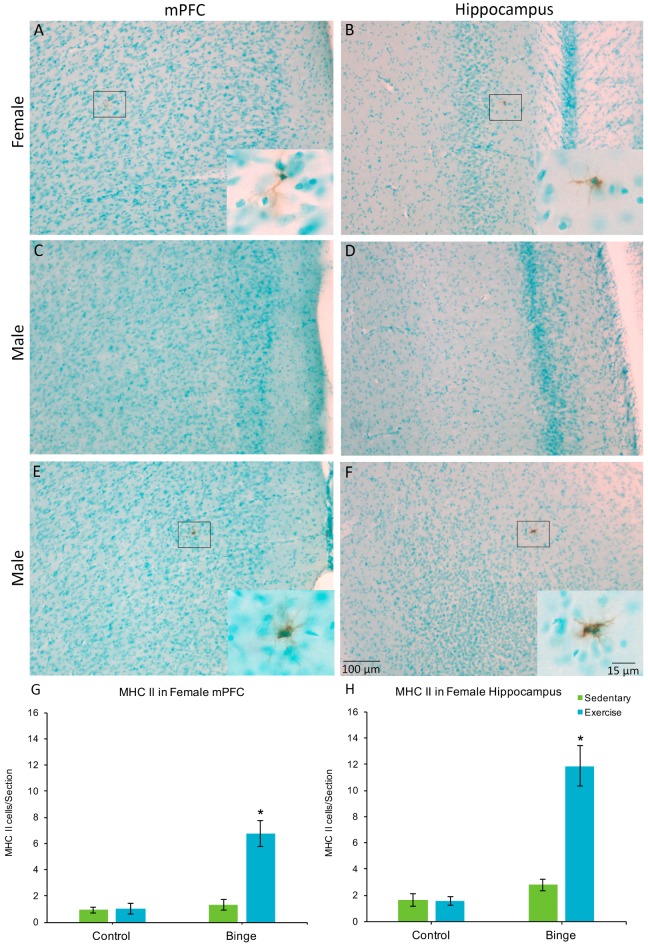
MHC II+ microglia were found in the mPFC (**A**) and hippocampus (**B**) of females but not males (**C**,**D**), with the exception of one sedentary binged male (**E**,**F**). In females, the combination of binge alcohol and exercise resulted in more MHC II+ cells in the mPFC (**G**) and hippocampus (**H**) compared to all other groups. Very few of the total microglia population expressed MHC II in either the mPFC or the hippocampus. * *p* < 0.001.

Only one male (in the BIN group) had MHC II+ cells, one in the mPFC and one in the hippocampus. None of the other males showed MHC II expression, therefore, males were not included in the statistical comparisons. In the mPFC, a factorial ANOVA revealed a Diet × Activity interaction for average number of MHC II+ cells per section (*F*(1,20) = 20.37, *p* < 0.001), Figure 4G). Post hoc pairwise comparisons show that the BEX females had significantly more MHC II+ cells than all other groups (*p*’s < 0.001). Similarly, for the hippocampus, there was a significant Diet × Activity interaction (*F*(1,20) = 29.68, *p* < 0.001) for the average number of MHC II+ cells per hippocampal section (Figure 4H).

## 4. Discussion

The effects of alcohol differ between the sexes, with females apparently more vulnerable to the negative, particularly the neurotoxic, effects [6,9,15]. The mechanisms underlying this apparent vulnerability of the female brain to alcohol-induced damage have not been widely investigated, although dysregulation of trophic support, stress hormones, astrogliosis, and inflammation have been implicated [15,56,57,58]. Sex differences in the microglial response to alcohol may be a contributing factor, so in the current study we quantified microglial number and morphology following binge alcohol and/or exercise. Our hypothesis was that binge alcohol would prime microglia to react to a second stimulus—exercise. We also hypothesized that this effect would be stronger in females. We found that binge alcohol did in fact induce microglia to adopt morphology consistent with priming or partial activation, but only in females. Moreover, we did not find that post-binge exercise had any effect on the number of microglia or primed microglia in either sex. We did find that binge and exercise increased the number of MHC II+ microglia in females, but since the overall number of MHC II+ cells was quite low, we do not regard this as strong evidence that exercise acted as a secondary stimulus. 

Following binge alcohol, we found a decrease in microglia in the mPFC in males, but not females. In the hippocampus, there was a significant increase in microglia in binged females compared to males. Together, these results indicate that binge alcohol selectively increased microglia number in the female hippocampus but decreased it in the male mPFC and hippocampus. This increase in microglia number in the female hippocampus following the 4-day binge may be due to females mounting greater immune responses compared to males [16,17,18,19,20]; thereby resulting in females reactively generating more microglia in response to binge alcohol compared to males. Wilhelm and Hashimoto [57] found a similar dichotomous pattern of astrocytic activation following alcohol between the sexes: alcohol increased glial fibrillary acidic protein (GFAP) expression in the hippocampus of females but decreased it in males. These findings, coupled with the results of the current study suggest a sex difference in the glial response to alcohol, in which alcohol exposure increases glial cells in females but decreases them in males. However, increased microglia proliferation has been found following the 4-day binge in the hippocampus and cortex of male Sprague-Dawley rats using BrdU and Iba1 labeling [59]. In the current study, we found the number of microglia decreased in the mPFC and hippocampus of males following binge alcohol. However, BrdU labeling was not used to distinguish microglial cells generated after the binge. Therefore, it is possible that the BIN males in the current study had more reactively generated microglia than the males given the control diet. Future studies are needed to fully examine the mechanisms and functional implications of the sex differences in the glial response to alcohol. 

Although the change in microglia number found in the current study demonstrates a sex difference in the microglial response to alcohol, it is difficult to assess functional ramifications based on microglial number alone. Microglia are the immune cells of the brain, and as such are implicated in nearly every neurological disorder. However, microglia are also crucial for maintaining healthy neural functioning [45,55,60,61]. Therefore, the number of microglia alone is not as telling as their functional state. Prior studies have found a higher percentage of microglia with a partially activated or primed morphology in adult females compared to adult males [16,32,33,34]. The current study extends these findings by demonstrating sex differences in microglia morphology following binge alcohol. In the mPFC and hippocampus, BIN females had the highest number of microglia with thick arbors and large somas, morphological characteristics consistent with priming or partial activation [34,35,48,55]. Additionally, these features are consistent with the binge-induced alterations in microglia morphology we have found previously in females [40]. This increase in overall microglia number and priming in females may contribute to the apparent sex differences in alcohol effects and may have implications for long-term outcomes, as primed microglia launch exaggerated responses to secondary challenges including infection, injury, and additional binge episodes [38,48]. 

MHC II is a hallmark of microglial priming, as its upregulation enables them to act as antigen presenting cells (APCs) in the CNS and elicit T-cell activation [45,46]. Therefore, MHC II is often used as a marker of primed microglia [47,48,49,50,51,52]. In the current study, we found MHC II expression only in one male brain (from the BIN condition). However, we found MHC II expression in all female brains, with BEX females showing a significant increase in MHC II+ cells in both the mPFC and hippocampus. The lack of MHC II in males following the 4-day binge is consistent with previous reports, which also failed to find any markers of classical activation in males following the 4-day binge, including TNF-α and ED1, but did find increased expression of the anti-inflammatory cytokine IL-10 and the growth factor TGF-β [38,62,63]. These results suggest the microglia in males following a single 4-day binge may be beneficial to the recovery process. The presence of MHC II in females could indicate that microglia become more activated in females after the 4-day binge compared to males, however there were few MHC II+ microglia per section compared to the number of morphologically primed microglia. Therefore, most cells that displayed the partially activated morphology were not expressing MHC II. It is not known whether the microglial cells with partially activated morphology but no MHC II expression are pro- or anti-inflammatory. It is also important to note that the group with the most MHC II+ microglia were the binge exercise females, which suggests that a second stimulus was needed to increase MHC II expression. Future studies should determine which cytokines and growth factors are expressed by microglia following binge alcohol in males and females. It is possible that males and females both have an upregulation of growth factors and anti-inflammatory cytokines after a single binge. However, the slight expression of MHC II in females does suggest that a sex difference in microglial activation is possible. The number of MHC II+ microglia was small; however, this was only after a single binge, and binge drinkers do not binge only once. Subsequent binge drinking episodes may continue to increase the MHC II expression in the potentiated microglia. Additionally, since females started off with more morphologically altered microglia and MHC II expression, the rate of progression may be faster in females compared to males. The increased reactivity of microglia in females may contribute to the increased vulnerability to the neurotoxic effects of alcohol. However, future studies are needed to fully classify the expression profiles of the microglia in both sexes.

Based on our prior findings [40], we hypothesized that exercise would act as a secondary stimulus, such that the combination of binge alcohol and exercise would increase the number of microglia with primed morphology and MHC II expression, and that this effect would be stronger in females. This hypothesis was partially supported, as the combination of binge alcohol and exercise selectively increased MHC II expression in females. It is possible that this finding is due to the females in the present study running significantly further than males, consistent with prior studies [64,65]. This is unlikely, however, given that we found virtually no MHC II expression in males, regardless of exercise. Moreover, we found no evidence that exercise following binge alcohol increased microglial numbers or morphological evidence of priming in either sex. Consistent with our prior findings [40], we did observe that binge alcohol suppressed subsequent exercise-driven microglial plasticity in the mPFC, as exercise increased microglia number in control animals, but not binged, regardless of sex. A better understanding of the interactive neural effects of alcohol and exercise is crucial, as exercise is increasingly being used in the treatment of AUD [66,67,68]. While exercise is a low-cost means by which to enhance brain health, its optimal use for the AUD brain is dependent on determining the nature and extent of alcohol effects on subsequent exercise-driven neuroplasticity. The results of the present study suggest that binge alcohol may dampen exercise effects on microglia, but do not indicate that exercise acts as a secondary stimulus that elicits an exaggerated response from microglia primed by binge. 

It is possible that the observed sex differences in alcohol effects on microglia in the present study are due to males receiving overall less alcohol. During the 4-day binge, males had on average higher behavioral intoxication scores compared to females, which resulted in them receiving a lower average dose. The difference in behavioral intoxication ratings may be due to inherent sex differences in alcohol metabolism, as there is evidence suggesting that alcohol is eliminated faster in female rats compared to males [69]. Differences in alcohol metabolism (and therefore behavioral intoxication) between male and female rats may in part underlie the observation that in voluntary drinking paradigms, females drink more than males (reviewed in [70]). In the present study, despite the difference in alcohol dose, we found no difference in BEC or withdrawal symptoms. Moreover, binge alcohol increased the number of microglia in females, but decreased it in males. It seems unlikely that, had the male dose been matched to female, binge alcohol would reverse this relationship and result in an increase in microglia in the male brain. Nonetheless, the 4-day binge model used in this study was developed in male rats [53], and future studies of sex differences in mechanisms underlying alcohol-induced neurodegeneration may require a model utilizing fixed doses (for example [8]). 

## 5. Conclusions

Overall, the results from this study indicate a sex difference in the microglial response to binge alcohol, with females having significantly more microglia following binge compared to males, as well as having more microglia with morphology indicative of priming. The majority of these cells were not MHC II+ (immunocompetent), however, the morphological changes indicate that they are not unchallenged. These binge-induced microglial changes followed the same pattern in the mPFC and the hippocampus. If binge alcohol induces significantly more microglia to become partially activated in females compared to males, then females may have a larger reserve of microglia potentiated to launch an exaggerated response to subsequent challenges (such as another binge). This could contribute to the vulnerability of the female brain to the neurotoxic effects of alcohol. 

## Figures and Tables

**Table 1:** Experimental design.

Sex	Group	Timeline		
		4 Days	6 Days	11 Days
Females	CON	Control Diet	Rest	Sedentary
	CEX			Exercise
	BIN	Binge Diet	Rest	Sedentary
	BEX			Exercise
Males	CON	Control Diet	Rest	Sedentary
	CEX			Exercise
	BIN	Binge Diet	Rest	Sedentary
	BEX			Exercise

CON: control, sedentary; CEX: control, exercise; BIN: binge, sedentary; BEX: binge, exercise

**Table 2:** Scale of behavioral intoxication.

Intoxication Score	Indications	Dose (g/kg)
0	Normal rat	5
1	Hypoactive, mild ataxia	4
2	Ataxic, abdomen elevated	3
3	Ataxic with no abdomen elevation, delayed righting reflex	2
4	Loss of righting reflex, retains eye-blink reflex	1
5	Loss of righting reflex, loss of eye-blink reflex	0

Modified from Majchrowicz (1975).

**Table 3:** Withdrawal behavior scale.

Behavior Score	Indications	Behavior Score	Indications
0	Normal rat	2.6	General tremor
1.0	Hyperactivity	3.0	Head tremor
1.4	Tail tremor	3.4	Wet dog shakes
1.6	Tail spasticity	3.6	Chattering teeth
2.0	Caudal tremor	3.8	Convulsion
2.4	Splayed limbs	4.0	Death

Modified from Majchrowicz (1975).

**Table 4:** Binge data for males and females.

	Intoxication Behavior	*p*	Dose (g/kg/day)	*p*	BEC (mg/dL)	*p*	Median Withdrawal	*p*	Peak Withdrawal	*p*
Male	2.2 ± 0.1	0.01	2.9 ± 0.1	<0.001	485.6 ± 9.7	0.15	1.05 ± 0.2	0.27	2.7 ± 0.2	0.84
Female	1.5 ± 0.2		3.5 ± 0.1		460.07 ± 14.1		1.4 ± 0.2		2.8 ± 0.09	

**Table 5:** Exercise data for males and females.

	Average Distance (km/day)	*p*	Total Distance (km over 7 Days)	*p*	Peak Speed (km/h)	*p*
Male	1.1 ± 0.2	<0.001	11.4 ± 2.06	<0.001	16.2 ± 1.4	0.66
Female	2.7 ± 0.2		29.9 ± 2.7		17.1 ± 1.6	
